# Field-driven single domain wall motion in ferromagnetic nanowires

**DOI:** 10.1039/c8ra01597j

**Published:** 2018-04-18

**Authors:** L. D. Anh Ho, Minh-Tung Tran, Xuan-Huu Cao, Vinh-Ai Dao, Duc-The Ngo, Duc-Quang Hoang

**Affiliations:** Sustainable Developments in Civil Engineering Research Group, Faculty of Civil Engineering, Ton Duc Thang University 19 Nguyen Huu Tho Street, District 7 Ho Chi Minh City 700000 Vietnam hoangducquang@tdt.edu.vn; Faculty of Applied Sciences, Ton Duc Thang University 19 Nguyen Huu Tho Street, District 7 Ho Chi Minh City 700000 Vietnam; Advanced Program in Electronics & Communication Engineering, Da Nang University of Science and Technology 54 Nguyen Luong Bang Da Nang Vietnam; Department of Materials Technology, Faculty of Applied Sciences, Ho Chi Minh City University of Technology and Education 1 Vo Van Ngan, Thu Duc District Ho Chi Minh City 700000 Vietnam; Electron Microscopy Centre, School of Materials, University of Manchester Manchester M13 9PL UK

## Abstract

We present a Lorentz microscopy study of polycrystalline permalloy 2D nanostructures with a thickness of 20 nm. Each structure was designed as a single domain wall trap. The trap comprises two horizontal nanowires with an in-plane dimension of 200 × 1000 nm^2^, and three tilted pads with different shapes. These structures allow us to create head-to-head domain walls, and these created walls can propagate in the structures by an external magnetic field. These designed traps were simulated using the micro-magnetic OOMMF simulation software. Those nanostructures were also patterned using electron beam lithography and focussed-ion beam techniques. This aims to determine the geometric parameters required to propagate a single magnetic domain wall in these structures reproducibly. Among the studied structures with one and two field directions, we found that the motion of a domain wall can be reproducibly driven by two alternative field directions in a trap which consists of the two horizontal nanowires and three 90°-tilted ones. We investigated systematically the viability of both single field and sequential switching of two field directions. Lorentz microscopy and micro-magnetic simulation results indicate that the propagation of a domain wall is strongly affected by the precise shape of the corner sections linking the trap elements, and the angles of the horizontal nanowires and tilted pads. Domain wall pinning and transformation of wall chirality are strongly correlated to the trap geometries. Our results are vital to design an optimal trap which supports a reproducible domain wall motion. This might also support a greater understanding of domain wall creation and propagation in magnetic nanowires which are of interest for concepts of high-density and ultrafast nonvolatile data storage devices, including racetrack memory and magnetic logic gates.

## Abstract 2

A domain wall trap structure was found which is suitable for the field-driven single DW propagation between two narrow nanowires.

## Graphical abstract

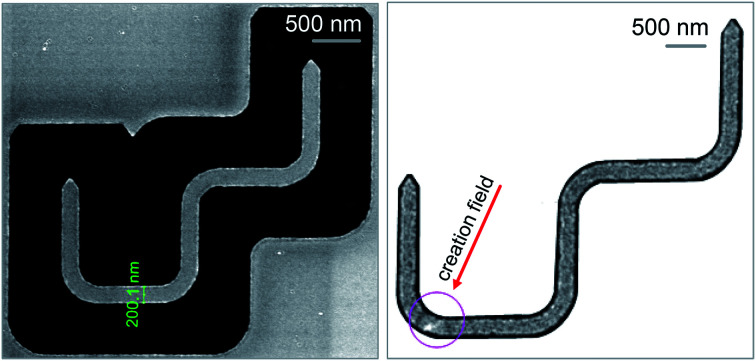

## 1 Introduction

Magnetic domain walls (DWs) in ferromagnetic nanostructures have attracted much attention due to their potential applications in spintronic devices such as domain wall logic gates and racetrack memory.^1–4^ An important parameter which directly affects the domain wall behaviour in such applications is the structural geometry, this can be engineered to either pin or allow the propagation of DWs in nanostructures.^1–10^ To achieve such devices in feasible applications, a complete understanding of structural geometry in connection with DW behaviour needs to be exploited. A preferable geometry is a domain wall trap (DWT) structure^5–7^ at which a DW can be created or driven to a particular position in the structure.^3–5^ These DWT structures were designed as discrete elements which consist of a narrow central section/nanowire. The two ends of the nanowire are connected with two wider sections/pads.

**Fig. 1:**
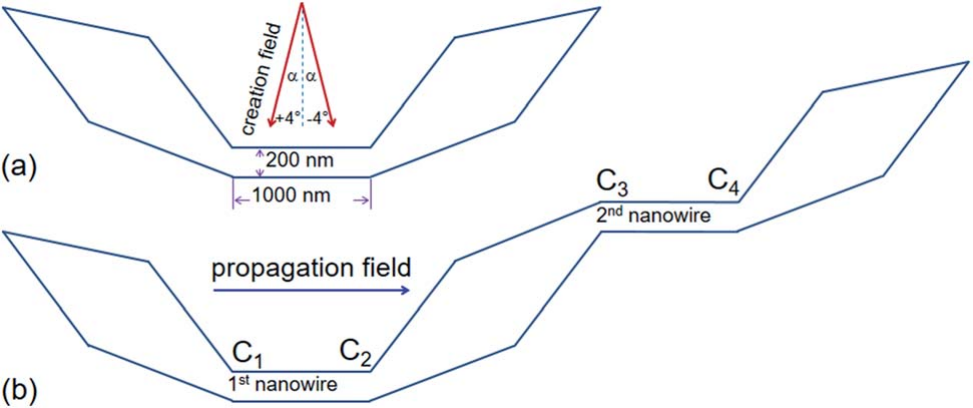
(a) A schematic of a domain wall trap (DWT) structure which consists of a narrow central section, the central section is linked to two wider end sections/diamond pads. Results of such DWT structures were reported in ref. 11. (b) Another DWT structure is considered in this work, it has two 20 nm-thick nanowires with the dimension of 200 × 1000 nm^2^ and three diamond pads. This structure also allows to create a single head-to head (H2H) DW at the first corner (C_1_), and the created H2H-DW can propagate from the first to the second nanowire under a propagation field, indicated as a rightwards arrow. Results of which are discussed in the text.

Among various geometries,^3–7^ a DWT structure which consists of a narrow nanowire with an in-plane dimension of 200 × 1000 nm^2^ and two wider sections or diamond shapes, is preferable. This structure proved most successful in terms of its ability to support a head-to-head (H2H) or a tail-to-tail (T2T)-DW. These DWs could be moved reproducibly with an external magnetic field, as seen in Fig. 1(a). This structure requires small fields to create and propagate a DW in the nanowire. This structure has a thickness of 20 nm, vortex DWs (VDWs) are frequently found in the central section. Herein, an external field was applied with a small angle (*α*) varying between ±4° in respect of the direction perpendicular to the easy axis of the nanowire.^3,5,11^ With this procedure, a DW can be created and driven to a particular position of the trap.^11^ Micro-magnetic simulation results of H2H DWs showed that both VDW and transverse DW (TDW) configurations can nucleate, depending on the width and thickness of the structure.^12–14^ While experimental results indicated that the asymmetry of the two-dimensional (2D) geometric structure might affect the magnetic configuration.^6,7^ The structure, as shown in Fig. 1(a), was also fabricated and characterized by Lorentz microscopy.^11^ Therein, an external magnetic field applied perpendicular to the easy-axis of the nanowire, a H2H/T2T VDW can create in the nanowire. Chirality of a VDW usually appears as counter-clockwise (CCW) or clock-wise (CW) which is recognized by darker or brighter core/spot in the Lorentz TEM image.^3,11^ An issue is being retained in this structure, how to propagate a DW from a nanowire to another one using an external magnetic field. In other words, an optimal DWT structure can be found at which it is suitable for field-driven DW motion through the optimal DWT structure, *e.g.* between two nanowires.

## 2 Structural designs and simulations

### 2.1 Structural designs

**Fig. 2:**
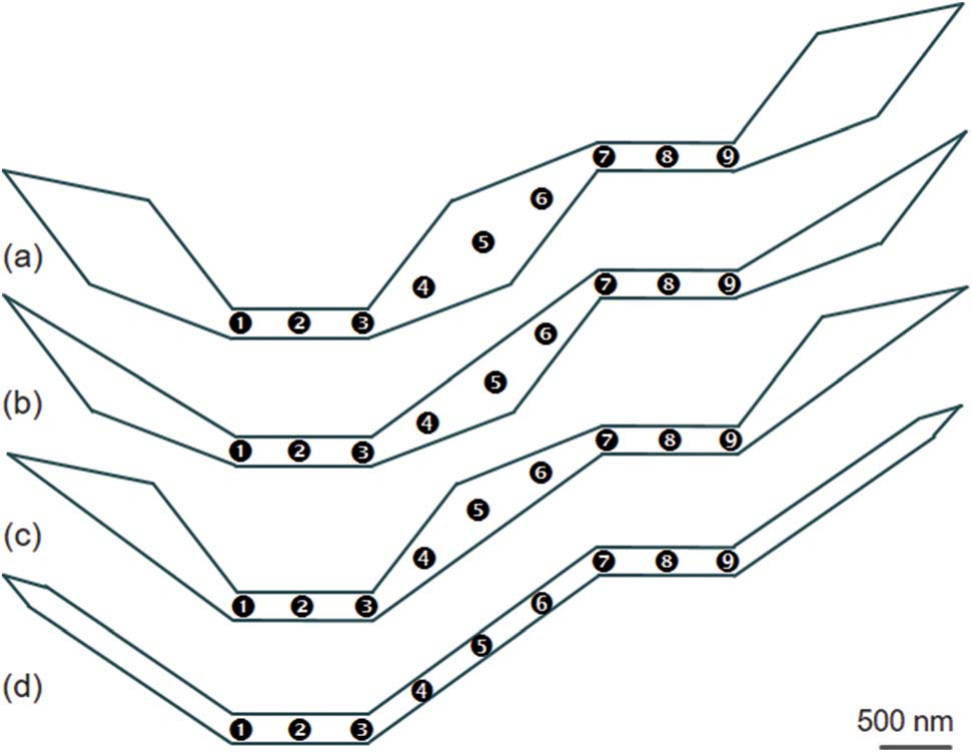
(a) A schematic of an original domain wall trap (DWT) structure which consists of two narrow nanowires with the in-plane dimension of 200 × 1000 nm^2^, linking to three diamond sections/pads. Three modified DWT structures at which the modified structures were cut out (b) the upper, (c) lower and (d) either side of the diamond pads. Nine positions between the two nanowire-ends are possibly considered, this aims to have a comparison in the DW propagation among these four DWT structures. Simulation and experimental results of those structures are discussed in the text.

To create a single DW instead of using an external field applies with the angle *α* in respect of the direction vertical to the easy axis of the DWT structure,^11,14^ scientists from various research groups have investigated DWT nanostructures with restrictions or protrusions,^15–20^ at which an injection pad was attached to one end of these structures. Such structures can provide a single H2H DW, created at the junction between the injection pad and the remaining part of the structure. With the use of an injection pad, a DW was successfully created in those DWT structures. The created DWs however did not move uniformly through these structures. The non-uniform propagation of the single created DW might result from the anisotropy of diamond shapes which act as potential barriers and/or wells. These structural characteristics resist the DW propagation at a certain degree. This might also come from the width of the narrow nanowire which plays as a restriction acting as a potential energy barrier. The role of the nanowire-widths in these DWT structures with an injection pad was characterized, and the results of which showed that a DW created by this method does not move producibly.^11,21,22^ An issue still remains in those structures, created DWs did not propagate uniformly through all the nanowires of these structures. To gain a deeper understanding on the role of each component in such structures, another DWT structure was designed, as originally depicted in Fig. 1(b). Three other structures were also designed, at which they were modified from the original structure, these four structures are shown in Fig. 2.

Fig. 2(a) shows the original designed DWT. The structure consists of the two narrow nanowires and three diamond pads. Nine positions between the two ends of these narrow nanowires will possibly consider, at which a created DW can be pinned at these locations in the DW propagation process. A H2H-DW can be created at the left-end of the first nanowire (C_1_). To understand how the structural geometry affects the DW propagation through these nine positions, those three diamond pads of the DWT structure are alternatively modified. Fig. 2(b–d) show three modified DWTs, these structures were cut out the upper, lower and either side of the diamond pads. In each structure, nine possible positions are also given, this aims to have a comparison in the DW propagation among those four structures. Those structures will be simulated using the OOMMF software,^5^ fabricated by electron beam lithography (EBL) and lift-off techniques,^21^ and characterized by the modified field emission gun transmission electron microscopy (FEG-TEM, CM20).^23^ This is also a suitable routine to create a DW by applying an external magnetic field from the CM20 microscope.

### 2.2 Magnetic simulations and Lorentz TEM characterization

**Fig. 3:**
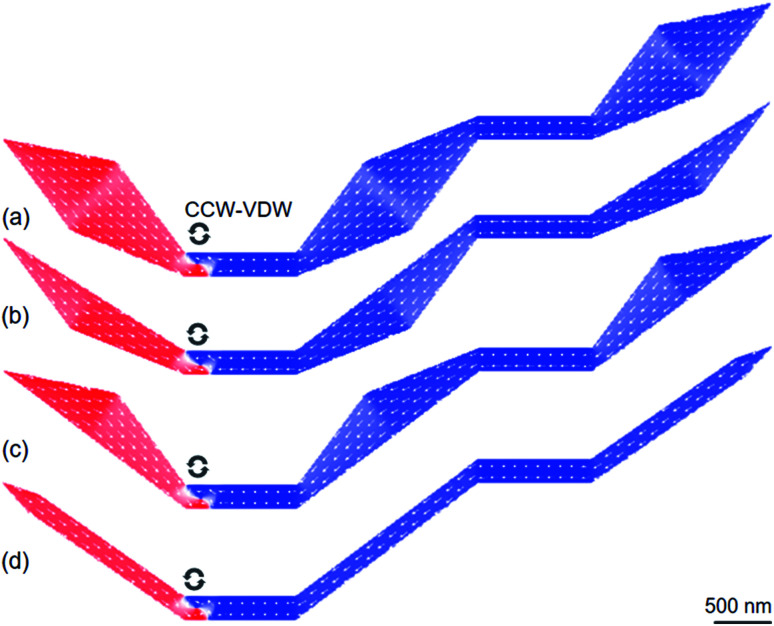
OOMMF images of (a) an original domain wall trap (DWT) and (b–d) its modified structures. Herein, spins are denoted as white tiny arrows and a CCW-VDW was intentionally created in each structure.

**Fig. 4:**
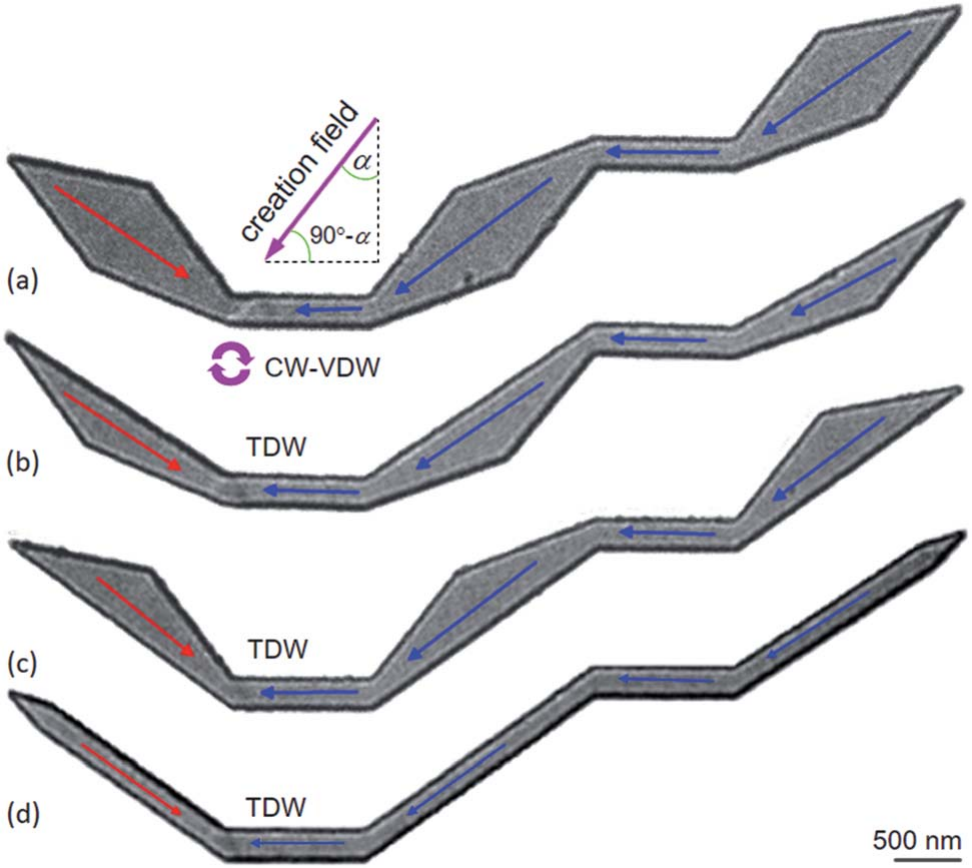
Lorentz images of the initial states of the DWT structures, as seen in Fig. 3, at which H2H DWs were created at the C_1_ corners of these structures using a creation field of 0.5 T applied with an angle of ∼40° (*α*). Based on the magnetic contrast of those images, magnetization in each sectional area of these structures is recognizable, *i.e.* magnetizations in either side of the created DW are denoted as red and blue arrows. Despite the DW creation procedures are similar for all the structures, the created DWs are however different in their wall types, *i.e.* (a) VDW, (b–d) TDW.

The designed structures were simulated with a cell-size of 10 × 10 × 20 nm^3^ using the OOMMF.^5^ The OOMMF simulations used the Landau–Lifshitz–Gilbert equations for the precession and damping of magnetization (*M*) under an external magnetic field (*H*) where *H* relates to the total energy (*E*_tot._) of the ferromagnetic system with a volume (*V*), *H* = −(1/*μ*_0_*V*) (∂*E*_tot._/∂*M*). The relation between *M* and *H* is, d*M*/d*t* = −*γ*_0_(*M* × *H*), where *t* is the time, *γ*_0_ is the gyromagnetic ratio, *μ*_0_ = 4π × 10^−7^ (V s A^−1^ m^−1^) is the exact magnetic permeability of free space. The parameters used for the simulations were, saturation magnetization, *M*_s_ = 8.6 × 10^5^ A m^−1^; exchange stiffness constant, *A* = 1.3 × 10^−11^ J m^−1^; magneto-crystalline anisotropy, *K* = 0; damping coefficient, *α* = 0.5. The initial states of all structures were taken to be H2H CCW-VDWs at the C_1_ corners of the first nanowires, as shown in Fig. 3. Therein, spins are denoted as tiny arrows. These created H2H-VDWs can propagate through the DWT structures with an application of the horizontal field. This sequence is achievable in experiments with the modified Philips CM20.^11,18,21–23^ Lorentz images of those patterned structures are also shown in Fig. 4, at which H2H DWs were intentionally created at the left-end corners of the first nanowires. In fact, DWs created in different types, *i.e.* VDW, TDW, in despite of the experimental procedures are the same for all the structures. Based on the contrast of those Fresnel images, magnetizations in either side of these created walls are recognizable, denoted as red and blue arrows.

Fig. 4 shows H2H-DWs which were created at the left-ends of the first nanowires (C_1_) in the patterned DWT structures. Herein, an external magnetic field of 0.5 T was applied with an angle of ∼40° (*α*) in respect of the direction vertical to the easy-axis of the nanowires. Despite these DW creation sequences are similar for all structures, those created wall types are different. A CW-VDW was created in the original DWT structure (Fig. 4(a)), whilst TDWs were formed in the other modified DWT structures. The discrepancy in these wall types might result from differences of spin configurations in either side of the created DWs, and potential energies at those corners.^18,21,24–28^

## 3 Experimental details

The 20 nm-thick DWT structures studied here were fabricated with two main steps: (1) a continuous 20 nm-thick Py film was evaporated onto an electron transparent TEM membrane using a thermal evaporator with the evaporation rate of 0.03 nm s^−1^.^29,30^ The TEM membrane is a 35 nm-thick amorphous Si_3_N_4_ film supported on a 500 mm-thick silicon frame with a 100 × 100 μm^2^ electron transparent window, obtained from TED PELLA, INC. The film thickness was controlled and monitored using a quartz crystal microbalance technique where a correlation between mechanical oscillation and resonant frequency is monitored. The use of the TEM Si_3_N_4_ membrane allows us to investigate magnetic properties of the patterned structures directly from the Lorentz microscope.^23^ (2) The DWT structures with different geometries, as shown in subsections 2.1 and 4.4, were patterned using the EBL and focused-ion beam (FIB) techniques.^21,29–33^ These facilities are available in the James Watt and Kelvin nanofabrication and characterization centres at University of Glasgow. Using these fabrication methods, those designed structures were isolated from the continuous films.^21,29^ The magnetic characterization was performed using the Fresnel imaging mode of the FEG-TEM Philips CM20 with a defocus distance of 3.6 mm.^29^ To avoid charging during the EBL/FIB fabrication and TEM imaging, a very thin conducting Au layer of 10 nm was deposited in the backside of the TEM-Si_3_N_4_ membrane using a sputtering deposition. With the use of the EBL method, edge roughness behaviour of these DWT structures slightly improve as compared to that of structures fabricated by the FIB milling technique, this was partially discussed in other works.^29,30^

## 4 Results and discussion

### 4.1 Experimental results of the original and modified DWTs

**Fig. 5:**
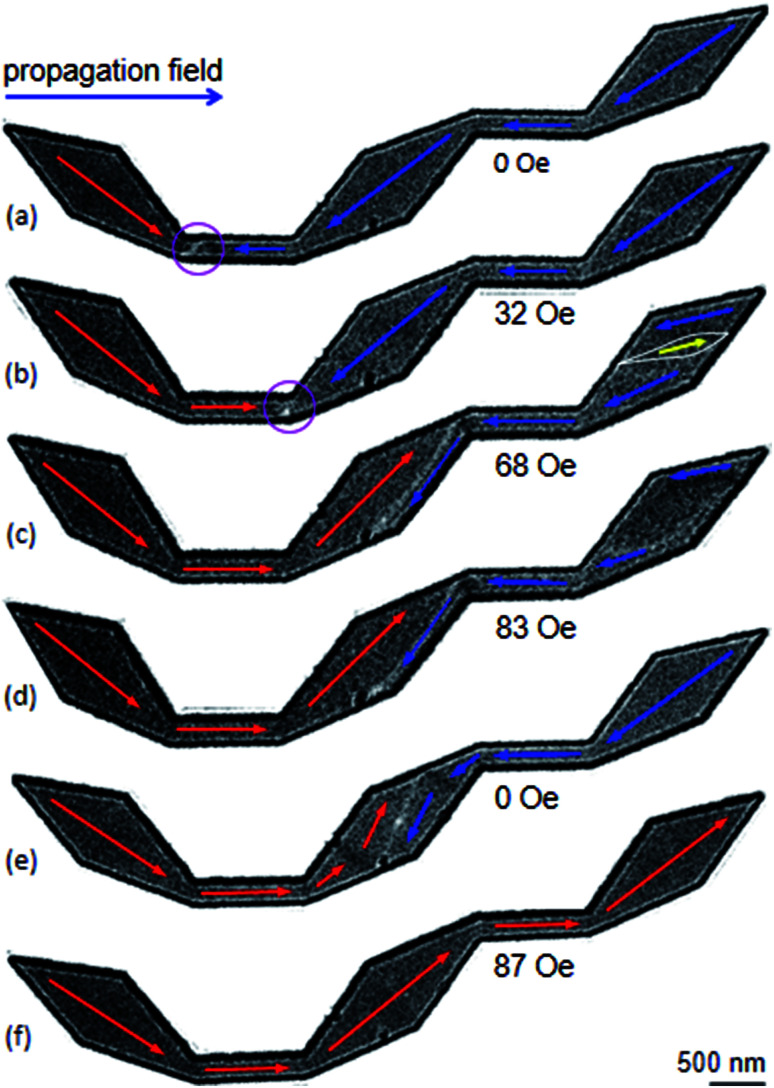
Lorentz images of the original DWT structure: (a) a CW-VDW was created by an external field of 0.5 T, the field was applied with an oblique angle of ≈40° (*α*), as also given in Fig. 4(a). (b) A Lorentz image of the DWT structure when the propagation field of 32 Oe was applied. (c)–(f) Lorentz images of the DWT structure which were recorded at various field strengths.

Experimental results obtained from those DWT structures (Fig. 4) will be discussed. Their OOMMF images are also given for a comparison. These simulations will assist the said experimental observations in interpretation. Fig. 5 shows Fresnel images of the original DWT structure at typical states. The propagation field strength was slowly increased in the direction parallel to the easy-axis of the structure. Fig. 5(a) shows a Fresnel image of the structure after the creation field of 0.5 T was applied. A CW-VDW was created at the C_1_ of the structure, as also seen in Fig. 4(a). At this state, the propagation field was applied in the horizontal direction, indicated as a blue arrow in Fig. 1(b). This aims to propagate the created DW from the C_1_ area to the end of the structure. The propagation field strength required to de-pin the created VDW from the C_1_ to C_2_ area is 32 Oe, *H*_depin_ = 32 Oe, Fig. 5(b). With an increase in the field to 68 Oe, the VDW entered into the connecting diamond pad, *H*_depin_ = 68 Oe, Fig. 5(c). The VDW is not only compressed onto one side of the connecting diamond pad, the spin orientation at the end-diamond pad is incrementally changed at *H*_depin_ = 68 Oe, as compared to the case at *H*_depin_ = 32 Oe, indicated by a yellow arrow inside an elliptical loop, Fig. 5(c). The size of this elliptical loop was enlarged when the propagation field strength was increased from 68 Oe to 83 Oe, Fig. 5(d). The VDW was trapped in the connecting diamond until *H*_depin_ = 87 Oe was applied to remove the wall out the structure, Fig. 5(f). The CW vortex chirality was clearly visualized in the structure when the propagation field was off, Fig. 5(e). The CW-VDW did not stop at any other locations in the remaining part of the structure, particularly in the second nanowire and the end-diamond pad. This hints that the motion of the created DW is strongly affected by the diamond pads of the DWT structure. Moreover, the created DW was strongly pinned at the connecting diamond pad. Therefore, the connecting diamond might plays as a potential energy barrier or well. Therein, it acts a protrusion associated with all the corners of the structure that prevent the DW propagation smoothly when the field is applied.^21,26,30^

As shown in Fig. 5(e), the VDW was trapped in the connecting diamond pad in the field range of 68–83 Oe. The coherent reversal process of spins is also visible at the end-diamond pad, indicated by a white-open ellipsoid. Therein, the spins in the ellipsoid are intentionally oriented to the field direction. The size of the ellipsoid increases when the field increases from 68 Oe to 83 Oe. We can conclude that the created VDW can propagate through the original DWT structure, and spins in each diamond pad rotate incrementally during the reversal process. Those reversal processes occurring in these diamond pads strongly affect the DW propagation in the structure.

**Fig. 6:**
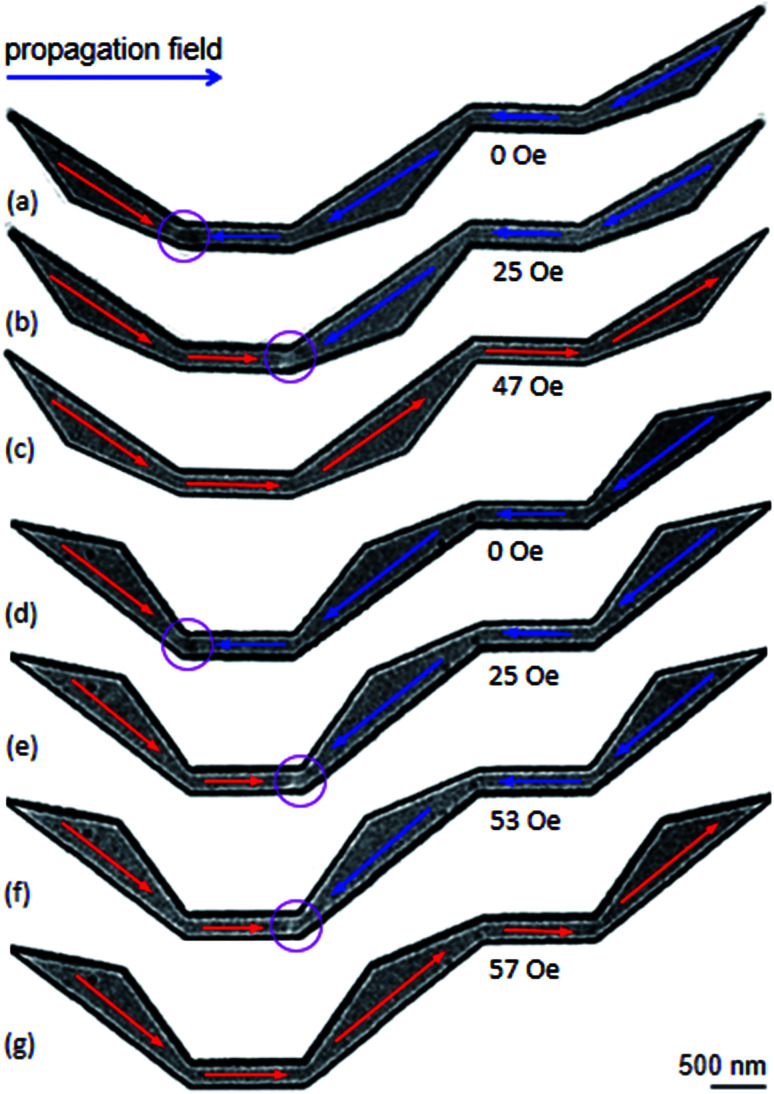
Lorentz images of the two modified DWT structures with the upper and lower sections of the diamond pads in those DWT structures were cut off: (a and d) TDWs were created at the C_1_ corners of these structures using a creation field of 0.5 T with *α* ≈ 40. (b and e) Lorentz images of the modified DWT structures when a propagation field of 25 Oe was applied. (c) and (f and g) Lorentz images recorded at different horizontal fields. These field strengths required to propagate or annihilate the created DWs from the structures.

Similar to the results observed in the original DWT structure, Fig. 6 shows Lorentz images of two other DWT structures at different field applications. The upper and lower sections of the diamond pads in those DWT structures were cut off. TDWs were created at the C_1_ corners of these structures, as seen in Fig. 4(b and c) and 6(a and d).

As seen in Fig. 6(a and c), the types of the created DWs in these two modified DWT structures are different from the wall type observed in the original DWT despite they were created in the structures with the same thickness. The field strength needs to propagate these created TDWs from the C_1_ to C_2_ corner, is of 25 Oe. This field value is lower than that required to propagate the created VDW in the original DWT structure, *H*_depin_ = 32 Oe. This difference might come from a difference in the effective surface areas of those structures. In other worlds, the de-pinning fields required to propagate a DW from the C_1_ to C_2_ in those three structures are in the range of 25–32 Oe. Those field values are comparable among those structures. When the propagation field increases to 47 Oe, the DW is annihilated in the DWT structure with the upper-diamond pad modification, Fig. 6(c), while the DW still remains at the C_2_ corner of the DWT structure with the lower-diamond pad modification at *H*_depin_ = 53 Oe, Fig. 6(f). This wall is strongly pinned at the C_2_, and de-pinned when the field strength of 57 Oe is applied, without stopping at any locations in the rest of the structure, *i.e.* C_3_, C_4_ and the second nanowire, Fig. 6(g). Particularly, Fig. 6(c and g) show a difference in the annihilation fields at the C_2_ corners of those structures, this might result from those C_2_ geometries and correlations of spin orientations in the two nanowires and modified diamond pads.

**Fig. 7:**
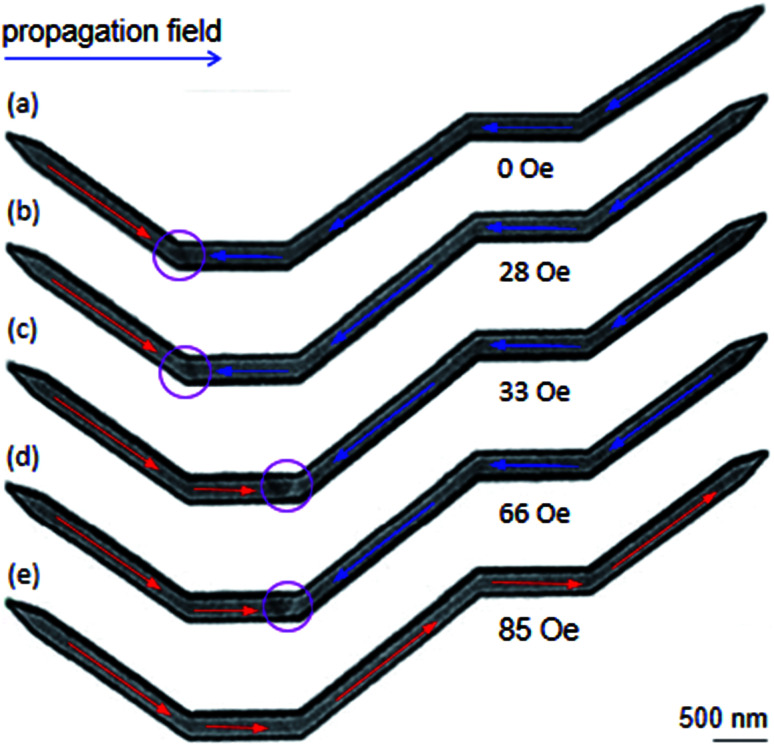
Lorentz images of the modified DWT structure where the diamond pads of the structure were modified in either side. (a) A TDW was created at the C_1_ corner of the structure with the creation field strength of 0.5 T (*α* = 40°). (b)–(d) The created TDW was propagated from the C_1_ to C_2_ corner with increments of the propagation field. (e) The TDW was removed out the structure at the field strength of 85 Oe.

To have a complete comparison, Lorentz images of the DWT structure with the diamond pads modified on either side, were recorded, as shown in Fig. 7. A TDW was also created at the C_1_ corner of the structure when the creation field of 0.5 T was applied, Fig. 7(a) or 4(d). This wall strongly pinned in the C_1_ corner even if the propagation field of 28 Oe was applied. When the propagation field of 33 Oe applied, the wall moved to the C_2_, and pinned at this area until the propagation field of 66 Oe was applied. Further increase in the propagation field, the wall removed completely from the structure at *H*_depin_ = 85 Oe. During the annihilation process, the wall did not stop at any locations in the remaining components of the structure. Based on the experimental results observed from the DWTs with and without diamond-pad modifications, which allow us to conclude that the created DWs could propagate from the C_1_ to C_2_ corner and unchanged wall chirality. These walls however did not propagate from the first to the second nanowire. Such propagation behaviour might result from a combination effect of the energy landscapes of all the corners, diamond/modified diamond pads and nanowires.

### 4.2 Simulation results of the original and modified DWTs

**Fig. 8:**
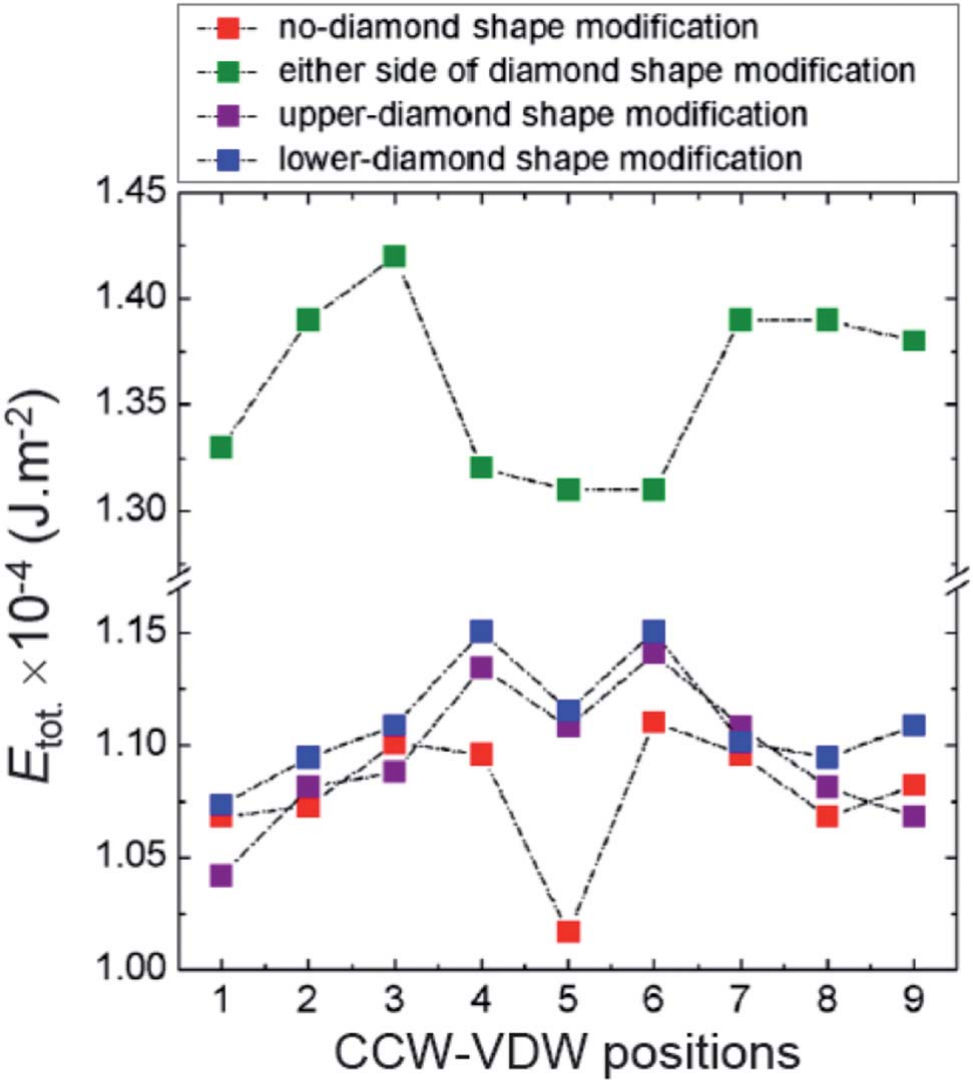
Normalized total energies of CCW-VDWs as a function of wall positions at the relaxed states, in which these CCW-VDWs were created at nine possible positions along the DWTs with and without diamond-pad modifications, as also seen in Fig. 2.

To explore the role of the connecting component of the DWTs on the above experimental results, CCW-VDWs were artificially created at nine possible positions in each of those structures, as depicted in Fig. 2. The total energies (*E*_tot._) of those VDWs were calculated. To bring the total energies of those structures at different states into comparison, the total energy values were normalized by their structural surface areas, results are plotted in Fig. 8.

As seen in Fig. 8, the energy landscape of the CCW-VDWs created in the DWT structure with the either-side diamond pad modification is higher than that of the other DWT structures, indicated as green symbols. Those three other structures show a similar trend in their energy landscapes. Particularly, the connecting components of the structures act as potential wells if a DW moves from the first to the second nanowire. The potential well width of the DWT with the either-side diamond pad modification is larger than that of the three others. This hints that if a VDW moves from the first to the second nanowire, it can be trapped in the connecting component parts of these structures. The C_2_ and C_3_ corners also act as potential barriers in the energy landscapes of these structures. This is fitted well to the case of the structure with the either-side diamond pad modification. While those barriers in the other structures are however shifted into the connecting part of the structures, *i.e.* no-, upper- and lower-modified diamond pads, as clearly seen in Fig. 8. Those offsets possibly result from the effective surface areas of the DWs created in the structure with the either-side diamond pad modification are equal in the connecting part and two nanowires. The DW sizes are however larger when they are created in the connecting parts of the structures with the no-, upper- and lower-modified diamond pads. Those created DWs were particularly created at the static states, therefore their potential energy levels could be approached to the metastable states. These barriers in the original DWT structure are lower than that of the other structures, indicated as red symbols. This implies that a DW can be easily trapped in the connecting diamond pad. This observation is in agreement with the experimental results, discussed in Fig. 5. Such characteristics of this DWT are fairly similar to the results observed by other authors when they considered a DW propagation in a straight or curved nanowire with protrusions.^18,26^ The DWT structures with the upper and lower diamond-pad modifications have a similar trend in their energy landscapes, at which the two potential wells at the position-➎ are still higher than the energies of the other states in either side of the connecting modified-diamond pads, *i.e.* locations: ➊ to ➌ and ➐ to ➒. While these minimum values of the other two structures are always lower than that of the positions in the two nanowires.

**Fig. 9:**
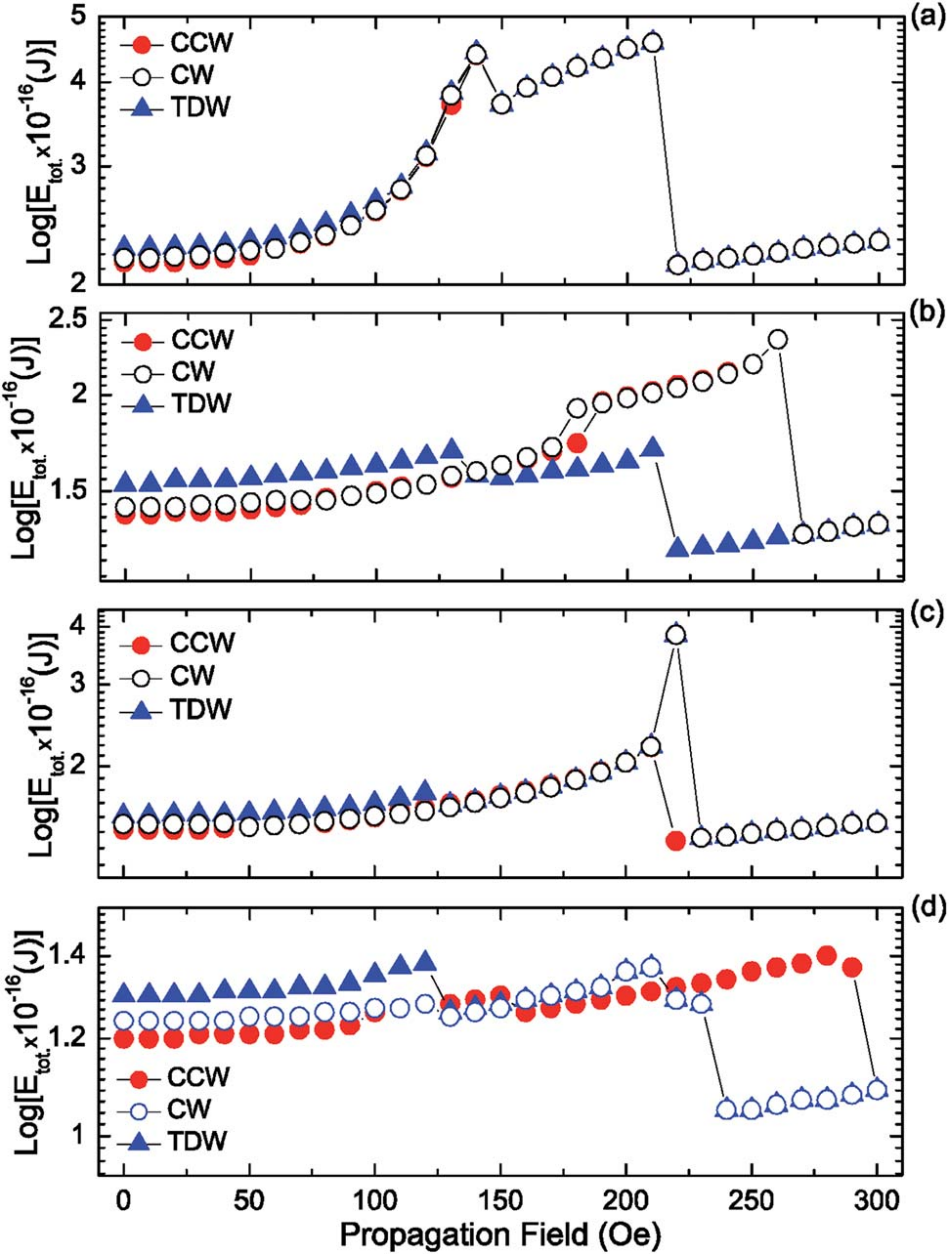
Total energies of the DWs created at the C_1_ corners of the DWT structures as a function of propagation fields, for the structures (a) without, (b) upper, (c) lower and (d) with either-side diamond-pad modifications. Therein, the red-solid-circle (-•-), black-open-circle (-○-) and blue-solid-triangle (-▴-) symbols represent the total energies of the CCW-VDW, CW-VDW and TDW, respectively.

In addition to the simulation results obtained from the relaxed states of the CCW-VDWs created at different locations in the DWT structures, a horizontal field was also applied to the DWs created at the C_1_ corners of those structures. Herein, the field strength was gradually increased with a step of 10 Oe. The total energies of these created DWs as a function of the propagation field, *E*_tot._(*H*_depin_), are plotted, and seen in Fig. 9.

Fig. 9(a) shows that the total energy landscapes of three wall types, *i.e.* CCW-VDW, CW-VDW and TDW, are mostly similar. In these *E*_tot._(*H*_depin_) curves, two energy barriers are recognizable at the propagation field strengths of 140 Oe and 210 Oe. This means that, when *H*_depin_ gradually increases, the created walls slowly move to the C_2_ corner and connecting diamond pad of the structure. The first energy barrier assigns at *H*_depin_ = 140 Oe as its come from the C_2_ corner and connecting diamond pad, whilst the other potential barrier assigns at *H*_depin_ = 210 Oe, this belongs to the C_3_, C_4_ corners and the end-diamond pad of the structure. At *H*_depin_ = 220 Oe, all of these walls were completely removed out the structure. The total energy levels of those walls are therefore unchanged afterwards.

Similarly, Fig. 9(b) shows the energy landscapes of those three walls which created in the DWT structure with the upper-diamond-pad modification. The *E*_tot._(*H*_depin_) curve of the created TDW (-▴-) is fairly similar to the results observed in the DWT without the diamond-pad modification. Therein, the *E*_tot._(*H*_depin_) curve consists of two potential barriers which are situated at *H*_depin_ = 130 Oe and 210 Oe. Whilst the other wall types, *i.e.* CCW and CW-VDWs, have a similarity in the total energy landscape, at which they have an only potential barrier at *H*_depin_ = 260 Oe. This hints that this DWT structure treats these two vortex walls in the same manner. Therein, the total energy slightly increases with an increase of the propagation field in the range of 0–170 Oe. This observation proves that those two walls were slowly pushed into the C_2_ of the structure. These walls are able to be rectified if they propagate through the structure with the upper-diamond modification. Hence, the more chances for the VDWs which can be trapped at the connecting modified diamond pad of the structure, while the TDW can be easily propagated through the DWT structure. The annihilation field requires to remove the TDW out the DWT structure is therefore low, *H*_depin_ = 220 Oe. In contrast, Fig. 9(c) shows that the energy landscapes of those wall types are also similar, at which they have an energy barrier at the field range of 210–220 Oe. The difference is that this structure rectifies the TDW and CW-VDW at a higher energy (*H*_depin_ = 220 Oe), whilst it needs a low energy for the CCW-VDW (*H*_depin_ = 210 Oe).

Fig. 9(d) shows that the energy landscapes of those three walls are different, this observation is in agreement with the results observed by other researchers.^27^ Therein, each wall type has its own energy at the initial stage, and those DWs can transform during the DW propagation.^29^ All the three wall types moved with their own energy levels to the C_2_ corner in the propagation field range of 0–120 Oe. The TDW and CW-VDW continuously propagated with the same manner in the remaining parts of the structure. However, the TWD moved with three different stages with a small difference in the total energy. Those stages are also visible in Fig. 9(d), with the field ranges are of 0–90 Oe, 100–150 Oe, and 160–290 Oe, respectively.

Based on the above results, we can assign that the DWT without the diamond-pad modification does not rectify those three wall types. Therein, a combination of the C_2_, C_3_ corners and the connecting diamond pad of the structure acts as a big potential barrier. This big barrier consists of two sub-barriers, and a small potential well at *H*_depin_ = 150 Oe. This implies that all of these walls can be trapped in this potential well. In contrast, the DWT structure with the diamond pads which were modified in either side, can rectify these three wall types at the low propagation field range of 0–120 Oe. These wall types moved with various energy levels in different stages of the propagation field. The differences in those energy levels are however small. This leads a fact that any wall propagating from one end to the other end of the structure can be trapped at locations between the two nanowires. In other worlds, the possibility of a DW trapping in the area between the two nanowires is higher than that in the DWT structure without the diamond-pad modification. The other two DWT structures with both upper and lower diamond pad modifications do not have such behaviour. This hints that it is difficult to have chances for a DW stopping at the area between the two nanowires. These simulation results are in agreement with our experimental observations obtained from those structures, as discussed in Fig. 5–7. Therein, the CW-VDW retains its chirality during the propagation from the C_1_ to C_3_ corner of the DWT structure without the diamond-pad modification, Fig. 5(a and e). The other structures can also retain the TDW configuration during the propagation between the C_1_ and C_2_, Fig. 6 and 7. A large difference in the energy levels of those DW configurations does not often mean that a DW can retain its chirality during the propagation process. In fact, many other parameters, *i.e.* spin orientations at the diamond or modified diamond pads, effects of edge roughness and structural geometries, could be involved in the DW propagation process. The potential energy landscapes are therefore unpredictable for each DW during the propagation process.

**Fig. 10:**
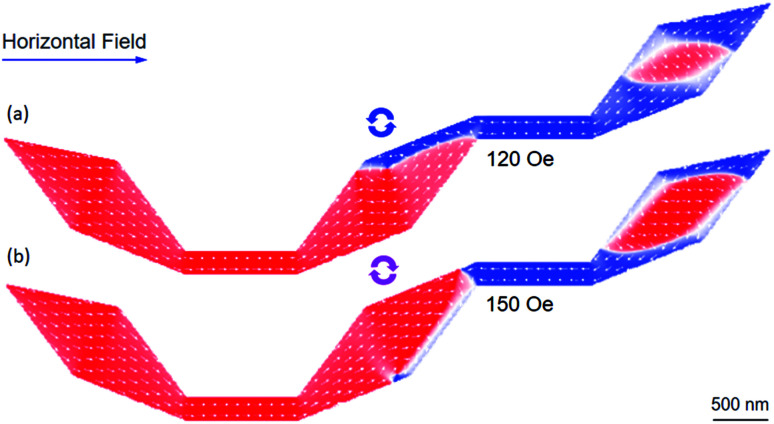
OOMMF images of two VDWs which were propagated to the connecting diamond pad of the DWT structure without the diamond-pad modification. These walls were initially created at the C_1_ corner. (a) The CCW-VDW was propagated from the C_1_ corner to the connecting diamond pad. Its chirality is maintained after the propagation through the C_2_ corner. Spins at the diamond end-pad were intentionally oriented to the horizontal field direction. (b) The CW-VDW also maintained its chirality during the propagation. Spins at the diamond end-pad were oriented similar to the CCW-VDW case. These coherent processes occurred in the connecting and ending diamond pads which were experimentally observed, Fig. 5(c–e).

As examples, Fig. 10(a and b) show that the CW- and CCW-VDWs are trapped in the connecting diamond pad of the structure without the diamond-pad modification. On the increase of the propagation field strength, a coherent magnetization rotation or a reversible process occurs in the diamond pad at the end of the structure. At the same time, the DW propagation could be induced by such processes, *i.e.* motion and reversal processes. A complex DW propagation process can be therefore obtained. When a field of 120 Oe was applied to the structure, this field pushed the CCW-VDW to the upper side of the connecting diamond pad. Whilst spins in the diamond end-pad were also oriented to the horizontal field direction, Fig. 10(a). Similar to the case of the CCW-VDW, when a horizontal field of 150 Oe was applied, this field pushed the CCW-VDW to the lower-right side of the connecting diamond pad. The coherent spin rotation also occurs in the end-diamond pad, Fig. 10(b). These coherent processes occurred in the connecting and ending pads of the structure which were experimentally observed, as discussed in Fig. 5(c–e). A difference in the field strengths between the experiment and simulation results is possibly resulted from a thermal effect which was excluded in the OOMMF simulation.^29^

**Fig. 11:**
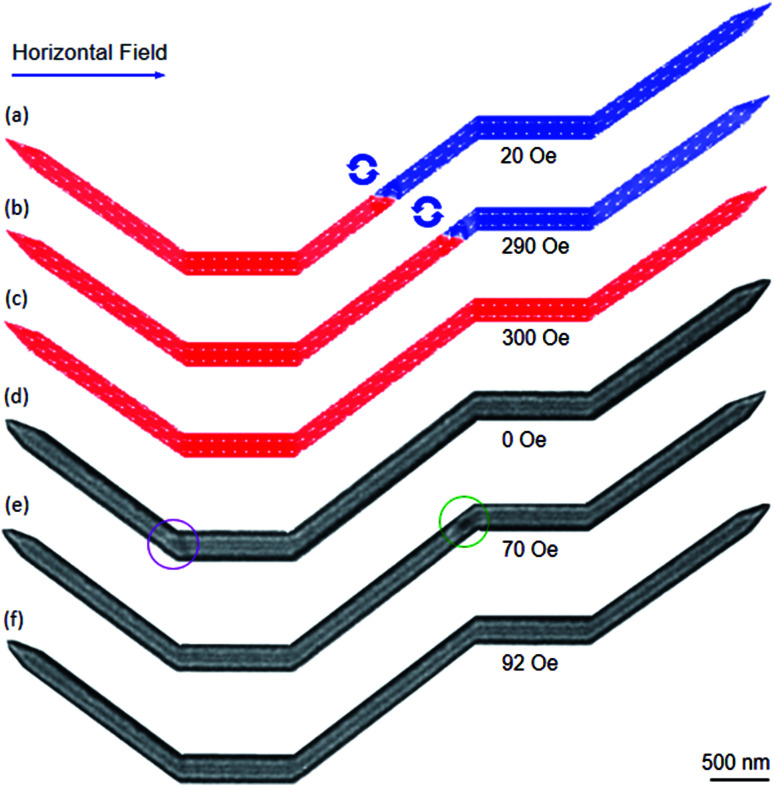
(a–c) OOMMF images of a CCW-VDW which was propagated in the DWT structure with the both upper and lower modified diamond pads, from the C_1_ to the connecting modified diamond pad of the structure. The wall propagated through the C_2_ under a horizontal field of 20 Oe, and its chirality also maintained. The propagation field strength was slowly increased to 290 Oe, the VDW incrementally propagated through the connecting modified diamond pad and closed to the C_3_. The wall was then removed out the structure at a field strength of 300 Oe, without stopping at any locations in the second nanowire. (d–f) Fresnel images of a TDW which was created and propagated in the given DWT structure. The TDW was strongly pinned at the C_1_. The wall propagated to the end of the connecting modified diamond pad (C_3_) when the horizontal field of 70 Oe was applied, and the TDW was transformed to a VDW. Further increase in the field strength, the VDW was annihilated at 92 Oe, without stopping at any locations in the remaining parts of the structure.

As shown in Fig. 9(d), the heights of the two maximum peaks at the low and high field ranges are comparable. The valley between the two maximum peaks is wider than that in the other cases. Such behaviour leads a DW can be easily trapped in the structure with the both upper- and lower-diamond pad modifications. In other words, we have raised the possibility to observe a DW which is trapped into the connecting modified diamond-pads, as compared to the other structures. Fig. 11 shows OOMMF and Fresnel images of a CCW-VDW and a TDW which were propagated in the DWT structure with the both upper- and lower-modified diamond-pads, from the C_1_ corner to the connecting modified diamond pad of the structure.

As seen in Fig. 11(a), the created VDW propagated through the C_2_ with a horizontal field of 20 Oe, and its wall chirality was maintained. This wall was slowly propagated through the connecting modified-diamond pad and closed to the C_3_ when the propagation field was increased in the field range of 20–290 Oe, Fig. 11(b). The VDW annihilated when a field of 300 Oe was applied, without stopping at any locations in the second nanowire, Fig. 11(c). Whilst a TDW was created at the C_1_ of the experimental structure, Fig. 11(d). The created TDW configuration might result from a combination effect of the structural edge roughness at the C_1_, the quality of evaporated Py, and spin orientations on either side of the TDW.^25–33^ Herein, the created TDW was strongly pinned at the C_1_ corner, Fig. 11(d), until the horizontal field of 70 Oe was utilized. The TDW moved to the end of the connecting modified-diamond pad (C_3_), and the TDW was transformed to a VDW, Fig. 11(e). Further increase of the horizontal field strength, the VDW was annihilated out the structure at a field strength of 92 Oe, without stopping at any locations in the remaining parts of the structure, Fig. 11(f). The coherence rotation processes were not clearly observed as visualized in the previous structures. This might result from the widths of the modified diamond pads that are relatively small as compared to the widths of their original diamond pads. Besides, the energy level of a CW-VDW configuration is always lower than that of a TDW. A TDW can be transformed to a VDW during the propagation process, due to defects, constrictions or protrusions.^17,24–30^ The CCW-VDW intentionally created at the C_1_ area in the nucleation process of the simulation, whilst the chirality of the created DW in the experiment largely depends on the nucleation field orientation and topological spin configurations at the C_1_.^27,34^

Field-driven single DW motion in the given DWT structures was investigated by means of OOMMF simulation and Lorentz microscopy studies. A single DW was successfully created in those structures, the DW propagation processes were studied systematically using a single propagation field direction. These created DWs are unable to propagate from the first to the second nanowire. The reason for this was found to be the connecting components and associate geometries at the corners. These parameters act as multiple potential barriers and wells. Such pinning potential landscapes will treat in different manners to those DW configurations during the DW propagation processes. A DW moves with a higher energy level which can transform to another wall type with a lower energy. The simulation and experiment results showed that the connecting components and corners of those DWT structures play a crucial role in the DW propagation processes. Another series of DWT structures was thus designed and simulated. This aims at understanding the role of those parameters.

### 4.3 One and two field-driven DW motions in DWT structures

**Fig. 12:**
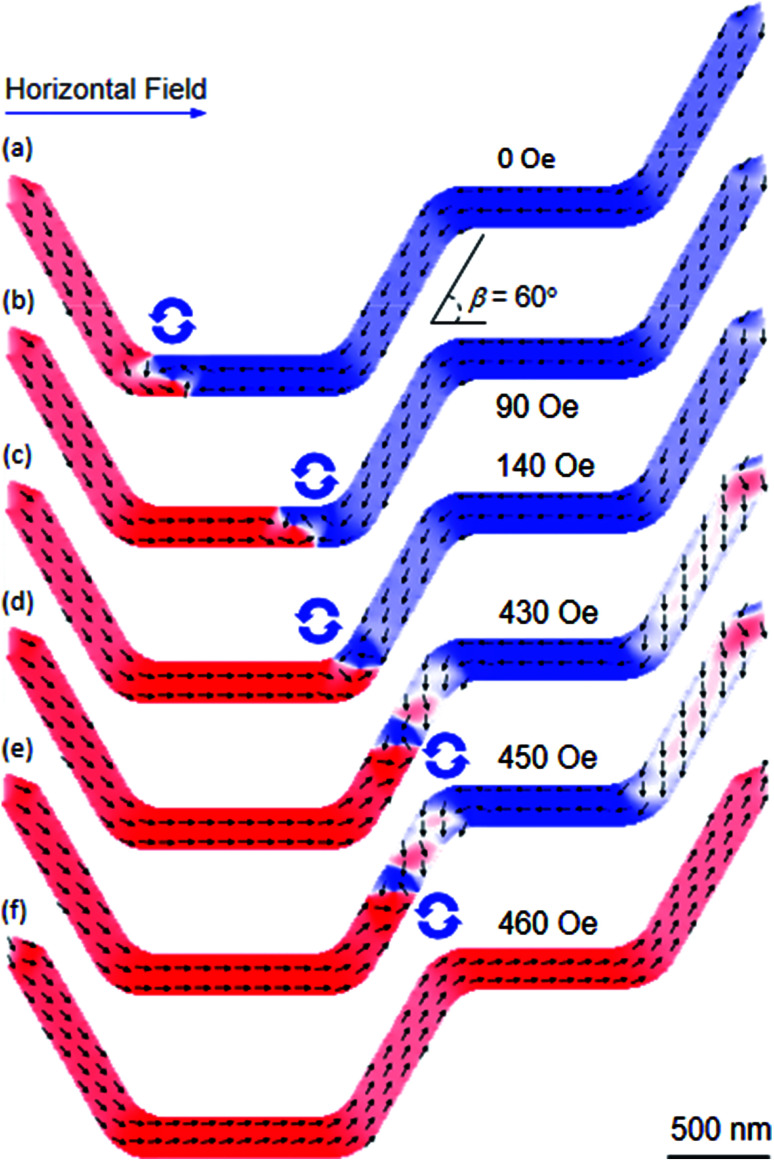
OOMMF images of a DWT structure which consists of the two horizontal nanowires, connected by another one. The connecting nanowire is tilted with an angle of 60° (*β*). (a) A CCW-VDW was created at the C_1_ corner of the structure. (b) The created VDW propagated to the end of the first nanowire under a horizontal field of 90 Oe. (c) The VDW was pushed into the C_2_ corner at *H*_depin_ = 140 Oe. (d and e) The VDW was slowly propagated along the connecting nanowire in the field range of 150–450 Oe, and spins were incrementally magnetized at the remaining parts of the structure, as visualized at the connecting and ending nanowires. (f) The VDW did not propagate to the second nanowire, and it was removed out the structure at *H*_depin_ = 460 Oe.

**Fig. 13:**
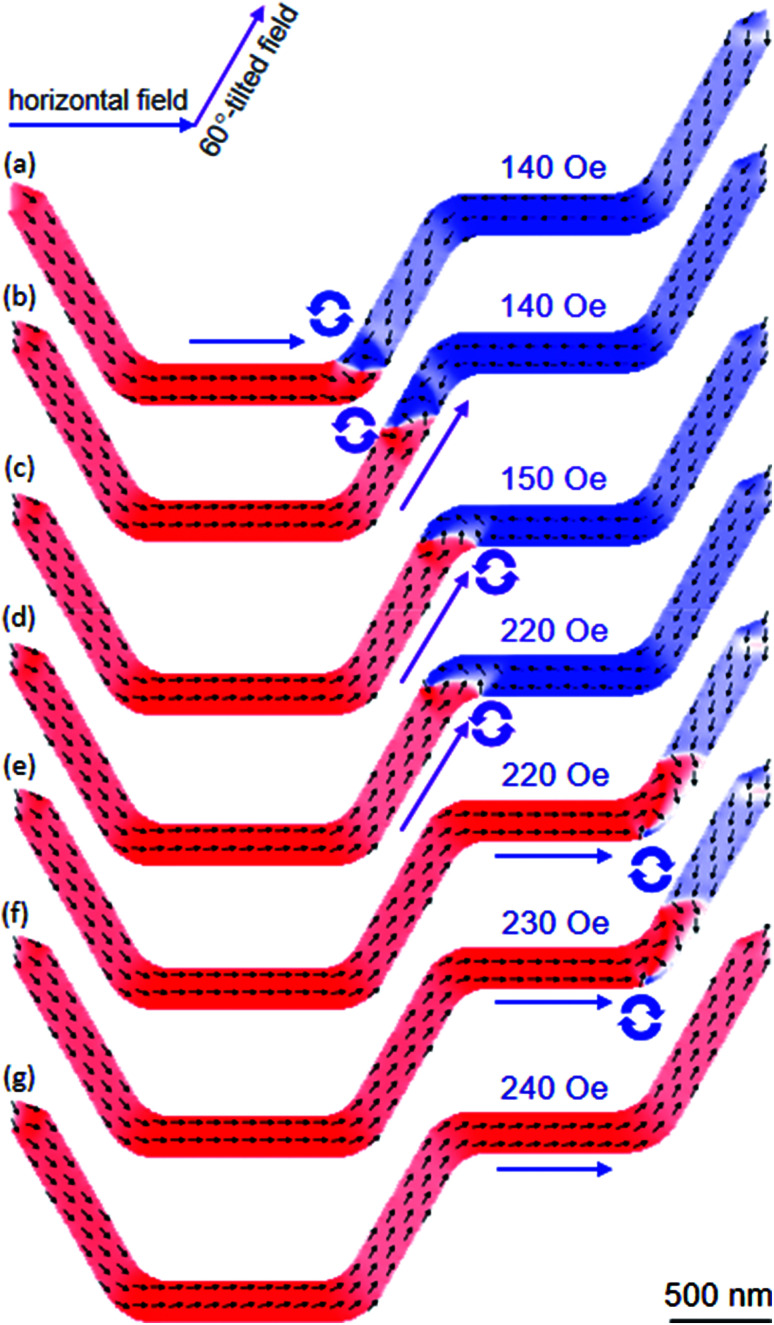
OOMMF images of the DWT structure, as given in Fig. 12, in which a combination of the horizontal and 60-tilted field directions was used. (a) The created CCW-VDW was propagated to the C_2_ under a horizontal field of 140 Oe. (b) Another field of 140 Oe was applied parallel to the easy-axis of the connecting nanowire. (c and d) The 60-field was incrementally elevated in the field range of 150–220 Oe, to push the VDW into the C3. (e and f) The 60-field direction was slowly rotated to the horizontal plane at *H*_depin_ = 220 Oe and 230 Oe, then the VDW was successfully moved to the end of the second nanowire. (g) The VDW was removed out the structure at *H*_depin_ = 240 Oe.

As mentioned, another series of DWT structures was designed and simulated. Therein, the corners of these structures were round off, and the angle (*β*) between the connecting components and nanowires varies from 15° to 90° with a step of 15°, one of those structures is given in Fig. 12, *β* = 60°. This structure was essentially modified from the DWT structure as shown in Fig. 11, at which the angle between the connecting nanowire and horizontal plane is of 60°, and the corners of the structure were also smoothed. This allows of a DW moving easily in the structure. A CCW-VDW was initially nucleated at the C_1_, as seen in Fig. 12(a). The VDW was moved to the C_2_ under a horizontal field of 90 Oe. When the horizontal field strength of 140 Oe was applied, the VDW moved into the right place of the C_2_. The horizontal field was elevated to 450 Oe, the VDW moved to the middle of the connecting nanowire. In particular, spins in the remaining parts of the connecting and ending nanowires were intentionally oriented to the horizontal field direction, as seen in Fig. 12(d and e). A further increase in the horizontal field strength, the VDW was removed out the structure at *H*_depin_ = 460 Oe without stopping at any locations in the second nanowire. This propagation behaviour is similar to the results observed from the DWT structure as given in Fig. 11. Based on these observations, even if the corners of the structure were smoothed, however we realized that the CCW-VDW did not propagate from the first to the second nanowire. We therefore make an attempt at applying an alternative field sequence, at which a combination of the horizontal and 60°-tilted field directions was applied alternatively to the structure, as described in the top-left corner of Fig. 13.

**Fig. 14:**
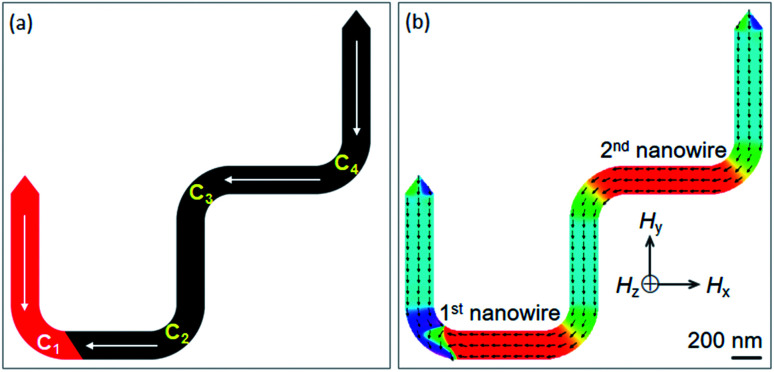
(a) A DWT structure was designed with *β* = 90°. (b) An OOMMF image of the 90°-DWT, at which a TDW was created at the C_1_ corner of the structure. Herein, the four corners (C_1–4_) of the structure were smoothed to assist a DW which can easily propagate through the structure using the two-field directions.

Similar to the procedure of the single field application, a CCW-VDW was propagated to the C_2_ corner of the structure at *H*_depin_ = 140 Oe, as seen in Fig. 13(a). The wall was continuously moved to the middle of the connecting nanowire at *H*_depin_ = 140 Oe when the 60°-tilted field direction was applied along the easy-axis of this nanowire, Fig. 13(b). The 60°-tilted field was slowly increased in the field range of 150–220 Oe, to push the VDW into the C_3_ corner, as shown in Fig. 13(c and d). The 60°-tilted field was slowly rotated to the horizontal plane at the same field (*H*_depin_ = 220 Oe), the VDW was successfully propagated to the C_4_ corner. The VDW was annihilated out the structure at *H*_depin_ = 240 Oe, Fig. 13(e–g). It is clearly that the field strength required to annihilate a DW out the structure with the two-field sequence method is much lower than that of the single field direction. Moreover, the CCW-VDW chirality retains until the DW propagates to the C_3_. The wall chirality changed to the CW-VDW configuration when it moved to the C_4_ with the two-field method. While the CCW-VDW chirality did not change in the case of the single field direction, as seen in Fig. 12. We can therefore conclude that with the use of the horizontal and rotational field directions alternatively, a DW can move between the two nanowires connected by another one. Nevertheless, the field strengths of these sequences are unequal in those nanowires. Among such designed structures, the DWT with *β* = 90° is able to satisfy our requirements, as shown in Fig. 14.

### 4.4 DW propagation in the 90°-DWT structure using the two-field directions

**Fig. 15:**
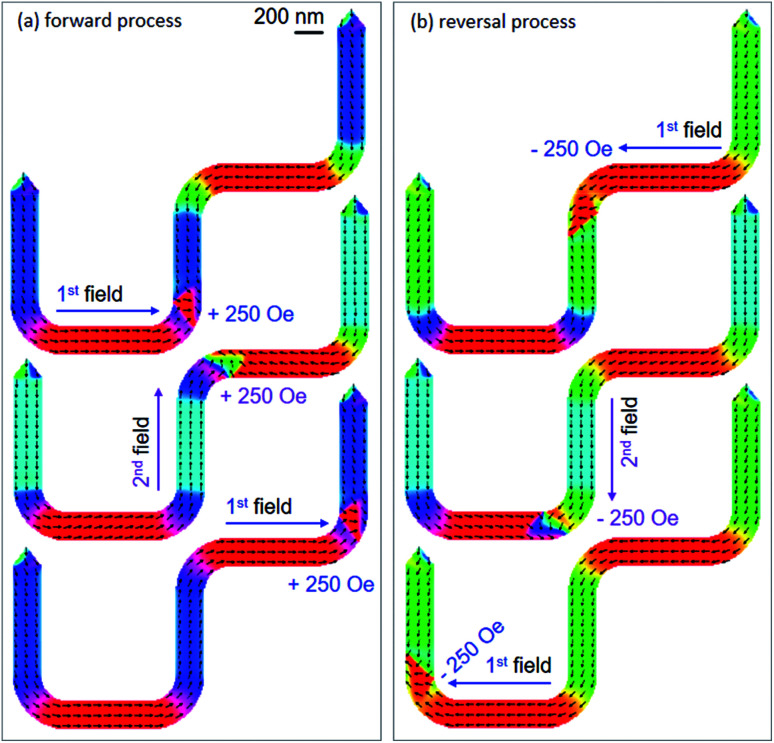
OOMMF images of (a) forward and (b) reversal processes of a TDW which was created, and propagated through the 90°-DWT structure (*β* = 90°) using the two-field directions. The field strength requires for each sequence is equal to 250 Oe.

As discussed, Fig. 14(a) shows the DWT which was designed with *β* = 90°. Therein, the four corners of the structure were rounded off. This aims to assist a DW which can propagate easily through the structure. This architecture might support the DW propagation between the two nanowires with the two-field method and an equal field value for each sequence can be achieved. Fig. 14(b) shows an OOMMF image of the structure with a TDW created at the C_1_. Even if a CCW-VDW was intentionally created, however a TDW was formed. This hints that the pinning potential landscape at the C_1_ plays an important role in the DW relaxation process. The created wall is actually constrained between the two 90°-magnetizations and located at the curved corner. Moreover, the effect of edge roughness is also an issue.^29^Fig. 15 shows OOMMF images of the TDW propagation between the two nanowires of the structure, at which the forward and reversal processes in a simulation are given in Fig. 15(a) and (b), respectively. The TDW was propagated to each corner of the structure with a field sequence of 250 Oe. It is clear to see that the propagation field requires equally for each sequence. This also means that the de-pinning field requires at each corner of the structure is more reproducible than that of the previous structures. In particular, the created TDW does not transform to other VDW types during the DW propagation process. This proves that the rounded corners play a significant role in the DW propagation processes.

Moreover, the spin rotations at the ending nanowire are not significant during the propagation process, as compared to the cases showed in Fig. 12(d and e). This might come from the easy-axis of the ending nanowire which is vertical to the horizontal field direction and/or the spin rotation process needs a higher horizontal field strength, *i.e.* >250 Oe. With a combination of these two-field directions and the given structural geometry, a TDW was successfully propagated from the first to the second nanowire, and *vice versa*. In particular, the de-pinning field strength is equal and reproducible for each sequence (*H*_depin_ = 250 Oe). This implies that the effects of edge roughness or pixelation at the corners of the designed 90°-structure are equal. The reversal processes of spins at the ending nanowire are also negligible, at which these parameters do not play a significant role in the propagation process in the field range of 0–250 Oe. To understand the role of structural characteristics at the corners and edges of the experimental DWT structure, the said structure was patterned using the FIB technique.^29–33^

**Fig. 16:**
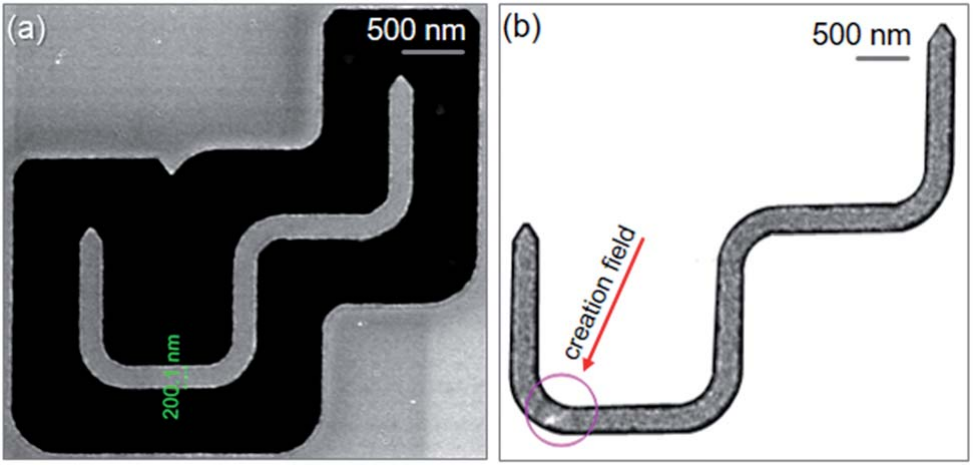
(a) A SEM image of the 90°-DWT structure which was designed, as given in Fig. 14(a), and patterned using the FIB method.^29^ (b) A Fresnel image of the patterned structure where a CW-VDW was created at the C_1_ corner using the creation field strength of 0.5 T with *α* ≈ 30°.

**Fig. 17:**
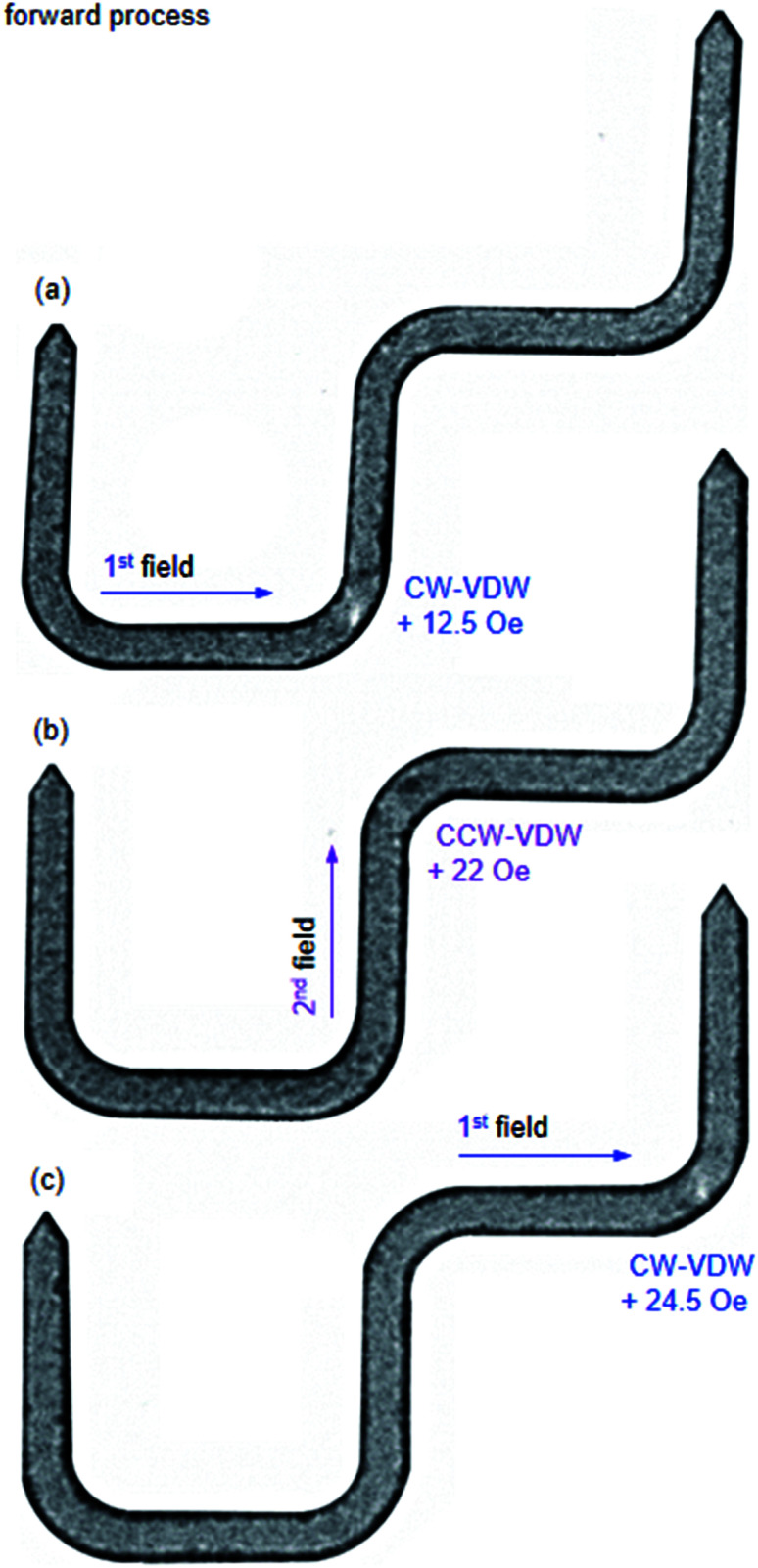
Fresnel images of the patterned 90°-DWT structure which were recorded at the three corners of the structure at different field strengths. (a) The created CW-VDW moved to the C_2_ with the first field direction, *H*_depin_ = 12.5 Oe. (b) The chirality of the VDW was transformed to the CCW-VDW type when it moved to the C_3_ with the second field direction, *H*_depin_ = 22 Oe, indicated as a dark spot. (c) The first field direction was applied again with *H*_depin_ = 24.5 Oe, to propagate the CCW-VDW into the C_4_ corner, and the chirality of the CCW-VDW was transformed to the CW-VDW, shown as a white spot.

Fig. 16 shows scanning electron microscopy (SEM) and Fresnel images of the patterned structure. The patterned structure is nicely isolated from the continuous 20 nm-thick Py film grown on a Si_3_N_4_ TEM membrane. Based on the contrast of the SEM image, the wire-width was measured approximately 200 nm. Therein, debris or re-deposition appears along the edges of the patterned structure indicating that the experimental structure was affected by the FIB irradiation processes, Fig. 16(a). This might affect the DW propagation in the structure with the two-field directions, *i.e.* domain wall behaviour at each corner. Magnetic characterization of the structure was carried out in the Lorentz TEM.^23,29,35,36^ A Fresnel image of the DWT structure was acquired, at which a creation field of 0.5 T was applied with *α* ≈ 30°, indicated as a red arrow in Fig. 16(b). Therein, a CW-VDW was created at the C_1_ corner, the VDW configuration slightly distorted and its vortex core shifted to the lower-edge of the corner, indicated as a white spot in Fig. 16(b). The chirality of the created DW depends on the field strength and *α*, those characteristics were studied by other authors.^6,7,11,21,37^ The two fields were alternatively applied to propagate the created VDW through the patterned structure. Fresnel images of these sequences in the forward process are given in Fig. 17.

Fig. 17(a) shows a Fresnel image of the structure, at which the CW-VDW propagated to the C_2_ using the first field direction, *H*_depin_ = 12.5 Oe. The chirality of the CW-VDW is maintained, indicated as a white spot at the C_2_ corner. The second field was used next, *H*_depin_ = 22 Oe, the CW-VDW was subsequently moved to the C_3_. However, the chirality of the CW-VDW type was transformed to the CCW-VDW one, indicated as a dark spot at the C_3_. The first field was applied again, to propagate the CCW-VDW into the C_4_, *H*_depin_ = 24.5 Oe. In particular, the chirality of the CCW-VDW returned to the CW-VDW. With the given results, we can conclude that the field strengths required to propagate a DW through the patterned structure are much lower than that used in the designed structure. This difference is possibly resulted from the effect of edge roughness in the patterned structure, at which the pinning potential landscapes at the long edges of the structure was significantly modified. The created DW could be confined in those potential modified landscapes, constituted by the shape anisotropy and magneto static energy at those corners and edges. Based on the above simulation and experimental results, a DW was successfully created and propagated between the two 200 nm-width nanowires of the 90°-DWT using the two-field method. Even if the field strengths required to de-pin a DW from the corners of the simulated structure are equal and reproducible, as seen in Fig. 15. However, the de-pinning fields need to push a DW out the corners of the patterned structure are different and much lower than that obtained from the simulated structure, as seen in Fig. 17. This discrepancy in these de-pinning fields that require to propagate a DW from the corners of the simulated and patterned structures might also result from a thermal effect. This effect was excluded in the simulation results, whilst the Lorentz TEM characterization was performed at room temperature.^29^

## 5 Conclusions

DWT structures were designed, simulated, patterned and made of Ni_80_Fe_20_. These structures mainly consist of two 200 nm-width nanowires and various connecting sections, were studied by means of OOMMF simulations and Lorentz TEM experiments. The DW propagations in those structures were systematically characterized. The propagation process of a created DW in each structure was mainly discussed. This study allows to explore a structure which is suitable for a DW propagating from the first to the second nanowire using an external magnetic field. Various structures with different geometries at their corners and connecting sections were exploited and found to be important. These parameters directly affect the DW motion in the horizontal field direction. From the results observed in the DWT structures using the single field direction, a new DWT structure was therefore designed with *β* = 90°. A suitable method was subsequently found to propagate a DW through the 90°-DWT structure, as a combination of two-field directions. The two-field directions were alternatively applied to the structure with an angle of those directions is of 90°. This sequence of the two-field directions will assist a DW which propagates between the two nanowires reproducibly in the simulated structure. A DW could also be propagated through the patterned structure. However, the field strengths required to de-pin a DW from the corners of the patterned structure are different. This discrepancy might result from orientations of local spins with respect to each field direction in a particular area of the patterned structure. The DW pinning behaviour and the transformation in the VDW chirality largely correlate to structural geometries, *i.e.* shapes of the corners, local spin orientations. Hence, our results will add into a new road map for the single DW motion in DWT nanostructures using both single and two-field directions. Herein, the DWT structure with *β* = 90° is suitable for the field-driven single DW propagation between two 200 nm-width nanowires *via* another 90°-tilted one. This also might support a greater understanding of the DW creation and propagation in ferromagnetic nanowires that are of interest to the concepts of magnetic high-tech applications.

## Conflicts of interest

There are no conflicts to declare.

## Supplementary Material
